# Associations between TyG-BMI and normal-high blood pressure values and hypertension: cross-sectional evidence from a non-diabetic population

**DOI:** 10.3389/fcvm.2023.1129112

**Published:** 2023-04-24

**Authors:** Nan Peng, Maobin Kuang, Yi Peng, Hang Yu, Shuhua Zhang, Guobo Xie, Guotai Sheng, Yang Zou

**Affiliations:** ^1^ Department of Cardiology, Jiangxi Provincial People's Hospital, Medical College of Nanchang University, Nanchang, China; ^2^ Jiangxi Cardiovascular Research Institute, Jiangxi Provincial People's Hospital, The First Affiliated Hospital of Nanchang Medical College, Nanchang, China; ^3^ Department of Emergency, Guangfeng District People's Hospital, Shangrao, China

**Keywords:** hypertension, TyG-BMI, general population, triglyceride glucose body mass index, normal-high blood pressure values

## Abstract

**Objective:**

Triglyceride glucose body mass index (TyG-BMI) has been shown to be strongly associated with a variety of chronic diseases. However, little is known about the associations between TyG-BMI and normal-high blood pressure (BP) values and hypertension (HTN).

**Method:**

The current study was cross-sectional in design and included 15,464 non-diabetic participants recruited between 1994 and 2016 in the NAGALA (NAfld in the Gifu Area, Longitudinal Analysis) study. Associations between TyG-BMI and normal-high BP values and HTN were assessed using multivariate logistic regression. The ability of the TyG index, BMI, and their combined index TyG-BMI to identify normal-high BP values and HTN was compared by receiver operating characteristic (ROC) curves.

**Results:**

Among the 15,464 eligible non-diabetic participants, 28.56% (4,416/15,464) and 6.23% (964/15,464) had normal-high BP values and HTN, respectively. Multivariate logistic regression analysis showed positive correlations between BMI, TyG index, TyG-BMI and normal-high BP values/HTN; after standardized regression coefficients, TyG-BMI had the strongest association with normal-high BP values and HTN compared to BMI and TyG index. In the fully adjusted model, the odds ratio (OR) value corresponding to the relationship between TyG-BMI and HTN/normal-high BP values was 2.35; when TyG-BMI was used as a categorical variable, compared with the lowest quartile of TyG-BMI the regression coefficient for the association of the highest quartile of TyG-BMI with normal-high BP values increased by 426%, while the regression coefficient for the association with HTN increased by 527%. In further spline regression analysis, we also found that there was a linearly positive correlation between TyG-BMI and systolic BP/diastolic BP (SBP/DBP), which supported the linear trend between TyG-BMI and HTN/normal-high BP values (*P*-trend <0.0001). In addition, ROC analysis showed that TyG-BMI had good diagnostic values for both normal-high BP values and HTN, and TyG index combined with BMI can significantly improve the ability of a single index to identify normal-high BP values and HTN.

**Conclusion:**

In the non-diabetic population, TyG-BMI showed a significant positive correlation with both normal-high BP values and HTN, and TyG-BMI was of higher value for the identification of both normal-high BP values and HTN compared to BMI and TyG index alone.

## Introduction

HTN refers to a common epidemic disease mainly characterized by increased systemic arterial BP, which can be accompanied by functional or organic damage to the heart, brain, and kidneys (1, 2), and is a major risk factor for cardiovascular disease and all-cause mortality (3, 4). According to the world's largest HTN research report, the number of adults aged 30–79 years with HTN increased from 650 million to 1.28 billion over the period 1990–2019 (5); however, nearly half of them are unaware that they have HTN (6). Furthermore, the proportion of patients with HTN who are optimally treated and controlled is low in the general population, especially in those who are in low-resource settings (6, 7). Normal-high BP value is a transitional stage between normotension and HTN; similar to HTN, it also would increase the risk of various cardiovascular diseases and bring a series of complications (8–10), but some studies have put forward different views (11). Overall, evidence based on prospective studies and meta-analyses indicated that the risk of developing chronic HTN in normal-high BP values patients was 2–3 times higher than that of normal people, and the risk of cardiovascular and cerebrovascular diseases was also significantly increased (12–14). Therefore, early identification of patients with normal-high BP values and reduction of potential risk factors to reduce the risk of developing HTN is necessary, which may be feasible and practical to reduce the burden of HTN-related diseases.

Previous studies have suggested that insulin resistance (IR) may be involved in the pathogenesis of HTN by mediating low-grade systemic inflammation (15–17). So far, researchers have proposed a variety of methods to assess IR, among which the gold standard test is the hyperinsulinemic-euglycemic clamp test (18), while the homeostatic model of IR (HOMA-IR) (19) is the most widely used indirect measurement method in clinical practice. However, considering the hyperinsulinemic-euglycemic clamp test is invasive and relatively time-consuming, expensive, and complicated (20), and HOMA-IR is easily affected by the accuracy of insulin measurement and has poor reproducibility (21). Therefore, the development of non-insulin-based IR surrogate indicators may be a more convenient, practical, and cost-effective approach, especially in primary care settings.

TyG-BMI, a recently developed IR surrogate, is an index composed of three easily measurable biochemical parameters [BMI, triglyceride (TG), and fasting plasma glucose (FPG)]. According to Er et al. TyG-BMI has a greater ability to identify IR compared to other non-insulin-based IR indices (22). Furthermore, in several recent epidemiological investigations, TyG-BMI was found to have excellent diagnostic value not only in identifying IR but also in assessing the risk of metabolic syndrome (MS), non-alcoholic fatty liver disease (NAFLD), HTN combined with hyperuricemia, pre-diabetes and diabetes (23–27). However, it remains unclear whether there is a similar relationship between TyG-BMI and normal-high BP values/HTN, and the diagnostic value of TyG-BMI for normal-high BP values and HTN in the general populations. To address these issues, this study was based on a large sample of 15,464 non-diabetic people in the NAGALA study to investigate the association between TyG-BMI and normal-high BP values and HTN, and to evaluate the accuracy of TyG-BMI in identifying normal-high BP values and HTN.

## Methods

### Data sources and study population

This study was a secondary analysis based on the NAGALA study to explore the associations of TyG-BMI with normal-high BP values and HTN. The NAGALA study is a chronic disease survey study conducted in Gifu Ward, Japan. Since 1994, NAGALA study has been continuously recruiting people who take part in health examinations in Murakami Memorial Hospital, aiming at investigating the potential risk factors of chronic diseases such as diabetes and NAFLD, so as to promote the development of public health. The detailed study design was carried out in a previously published article for illustration (28), and the available NAGALA study data have been uploaded to the Dryad public database by Prof. Okamura (https://doi.org/10.5061/dryad.8q0p192). According to the principle of open sharing of the Dryad database, all researchers can make full use of the datasets in it for in-depth exploratory analysis without violating the rights of the authors because the authors of the original data have transferred the ownership of the data to the Dryad database. Therefore, based on previous studies, the present study established several new research hypotheses based on the new research objectives: is there a correlation between TyG-BMI and normal-high BP values as well as HTN in the non-diabetic population? Is TyG-BMI more valuable in identifying normal-high BP values and HTN? In the current study, we extracted cross-sectional data from the NAGALA study from 1994 to 2016, continued to use the inclusion-exclusion criteria of the NAGALA study, excluding subjects with alcohol abuse, diagnosed liver disease and baseline medication, subjects whose FPG exceeded 6.1 mmol/L at baseline, subjects diagnosed with diabetes at baseline, and subjects with missing covariant data. In addition, 10 subjects who withdrew from the study for unknown reasons were also excluded, and finally, we included 15,464 subjects in the study analysis.

### Ethical approval and consent to participate

In the original study, the researchers obtained informed consent from each subject and the Ethics Committee of Murakami Memorial Hospital approved the previous study protocol (28). The current study was conducted as a secondary analysis of the NAGALA research data, and the Ethics Committee of Jiangxi Provincial People's Hospital reviewed and approved the current study protocol. The entire research process followed the Declaration of Helsinki.

### Data collection and measurement

As previously mentioned (28), the following information was recorded for each subject by trained investigators: sociodemographic characteristics (age and sex), lifestyle behaviors (smoking and drinking status, and habits of exercise), disease history (liver disease, diabetes), anthropometric parameters [weight, height, waist circumference (WC) and arterial BP], biochemical parameters [liver enzyme indicators (alanine aminotransferase: ALT, aspartate aminotransferase: AST, gamma-glutamyl transferase: GGT), blood lipid indicators (TG, total cholesterol: TC, high-density lipoprotein cholesterol: HDL-C) and blood glucose indicators (FPG, hemoglobin A1c: HbA1c)]. Blood samples for the analysis of biochemical parameters were collected at least 8 h after fasting and analyzed and determined by standard methods using an automated analyzer. Fatty liver was determined by experienced gastroenterologists based on the assessment of several sonograms under abdominal ultrasound, namely deep attenuation, hepatorenal echo contrast, vascular blurring, and liver brightness (29).

### Definition and calculation

BMI was calculated as weight/height^2^. Based on the BMI cut-off point for Asian populations proposed by the World Health Organization expert committee, we defined BMI ≥ 25 kg/m^2^ as overweight/obese and BMI < 25 kg/m^2^ as normal weight (30). TyG index = Ln [[FPG (mg/dl)/2] × TG (mg/dl)]; TyG-BMI = BMI × TyG index (22, 31). Smoking status was categorized into three groups based on subjects' smoking status prior to the baseline survey: non, past, and current; similarly, drinking status was categorized into never (<40 g/w), light (40–139 g/w), moderate (140–209 g/w), and heavy (>280 g/w) based on the subject's weekly alcohol consumption in the previous month (32); additionally, for habits of exercise, subjects who engaged in any form of physical activity at least once per week were considered to have an exercise habit (33).

### Definition of normal-high BP values and HTN

Normal-high BP values and HTN were defined according to the Japanese Society of HTN Guidelines for HTN Management (JSH 2019) (34). JSH 2019 defines office BP thresholds for HTN as SBP ≥140 and/or DBP ≥90 mmHg; normal-high BP values as SBP 120–129 mmHg and DBP <80 mmHg, or SBP 130–139 and/or DBP 80–89 mmHg.

### Statistical analysis

We first used HTN and normal-high BP values as outcome variables and TyG-BMI as dependent variables, respectively, and then included the other covariates one by one in the logistic regression model, and variance inflation factors were calculated to assess the non-collinear variables that could be included in the current applied model (Supplementary Tables S1, S2) (35). Subsequently, based on the Strengthening the Reporting of Observational Studies in Epidemiology statement, we established four multivariate logistic regression models to assess the association of TyG-BMI with normal-high BP values or HTN (36), and recorded the corresponding ORs and 95% confidence intervals (CIs). In the model adjustment of multivariate logistic regression, model 1 was adjusted for sex, age, and height; model 2 was further adjusted for fatty liver, the habit of exercise, drinking status, and smoking status based on model 1; model 3 was additionally adjusted for lipid glucose-related parameters (HDL-C, TC, TG, HbA1c, and FPG) based on model 2; and model 4 was adjusted for all non-collinear variables. Based on model 4, we further fitted the dose-response relationship between TyG-BMI and SBP, DBP by 4-knot natural smoothing splines. We also constructed ROC curves to evaluate the relative diagnostic strength of TyG-BMI for normal-high BP values and HTN, calculated the area under the curve (AUC) of TyG-BMI and its optimal threshold, and the comparison between AUCs was performed by the De Long test (37). In addition, exploratory subgroup analysis was also conducted in the current study. Based on Model 4, we evaluated the relationship between TyG-BMI and normal-high BP values/HTN in subgroups of age, sex, BMI, smoking status and drinking status respectively, and examined the differences of different stratification in subgroups by the likelihood ratio test.

All analyses in this study were performed on R language version 3.4.3 and Empower(R) version 2.0. Baseline data were grouped according to TyG-BMI quartiles, among which normally distributed continuous variables were expressed as mean ± standard deviation and compared between groups using one-way analysis of variance; meanwhile, non-normally distributed variables were expressed as median (interquartile range) and compared between groups using Kruskal-Wallis H test. Categorical variables were expressed as frequency (%), and differences between groups were compared using the chi-square test. We considered statistical differences to be significant when two-sided *P *< 0.05.

## Results

**Figure 1:**
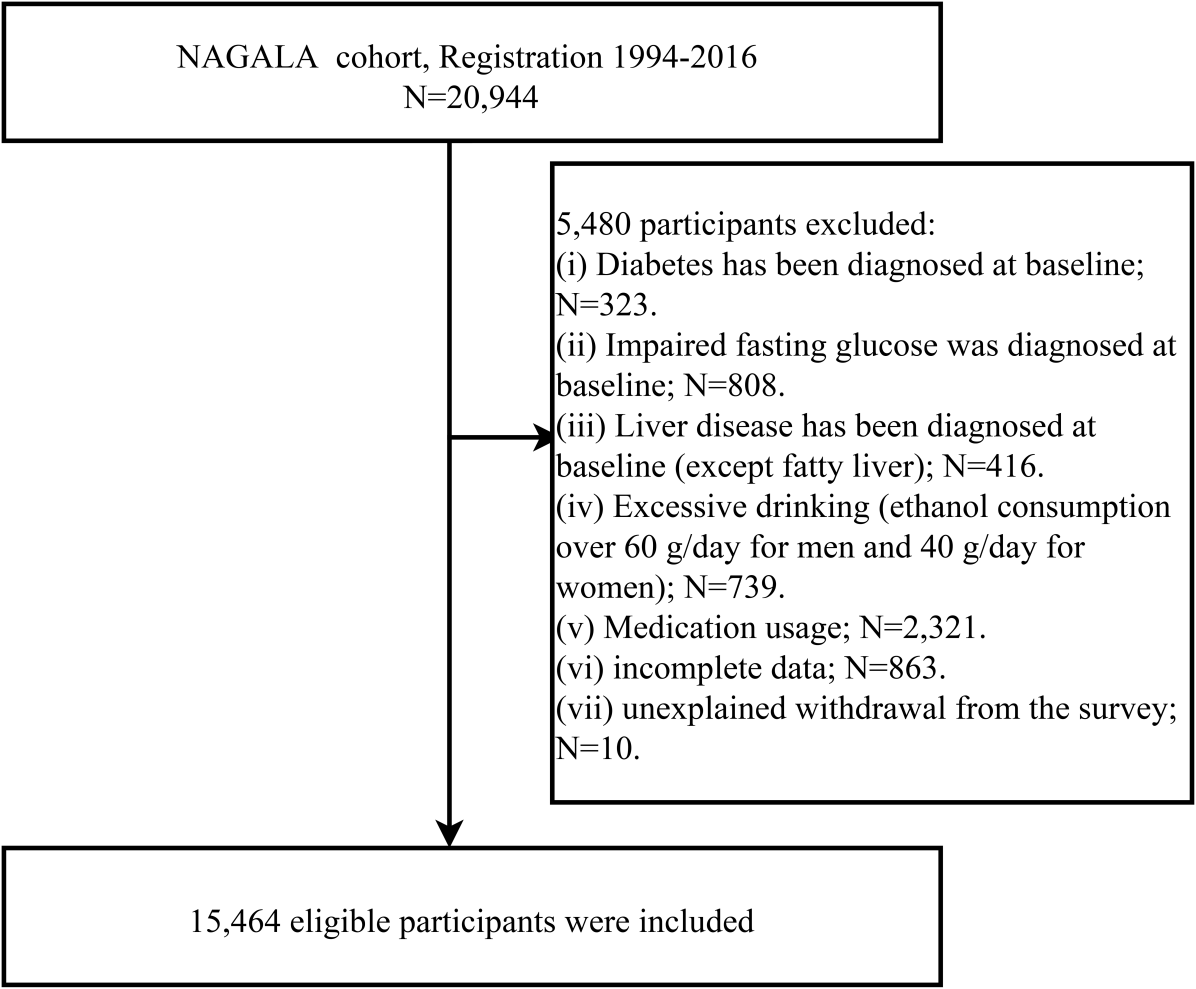
Flowchart of the selection process of study subjects.

Between 1994 and 2016, the NAGALA study recruited 20,944 people. A total of 4,339 subjects were excluded due to missing data (*n* = 863), known liver disease (*n* = 416), excessive drinking (*n* = 739), and oral medications at baseline (*n* = 2,321). In addition, in the baseline examination, 323 diabetic patients and 808 patients with FPG >6.1 mmol/L, and 10 subjects who withdrew from the study for unknown reasons were further excluded. Ultimately, 15,464 individuals were included in this study. The flow chart of subject selection was shown in Figure 1.

Table 1 shows the baseline characteristics of the study population according to the TyG-BMI quartiles. In the quartile with the highest TyG-BMI, the levels of age, weight, height, BMI, WC, ALT, AST, GGT, SBP, DBP, TC, TG, HbA1c, FPG, and TyG index were highest, but HDL-C had the lowest level. The prevalence of normal-high BP values in the highest TyG-BMI group was 11.67%, which was much higher than that in other TyG-BMI groups (Q1: 3.07%, Q2: 5.49%, Q3: 8.33%); similarly, in the highest TyG-BMI group (Q4), the prevalence of HTN was 3.55%, which was also much higher than that in other TyG-BMI groups (Q1: 0.27%, Q2: 0.82%, Q3: 1.60%).

**Table 1:** Clinical characteristics of the study population according to TyG-BMI quartiles.

	TyG-BMI quartiles				*P*-value
	Q1 (97.49–153.10)	Q2 (153.11–174.15)	Q3 (174.16–199.26)	Q4 (199.27–421.35)	
Participants(*n*)	3,866	3,866	3,866	3,866	
Age, years	39.00 (35.00–45.00)	43.00 (37.00–50.00)	44.00 (38.00–52.00)	44.00 (38.00–51.00)	<0.001
Sex					<0.001
Men	887 (22.94%)	1,788 (46.25%)	2,678 (69.27%)	3,077 (79.59%)	
Women	2,979 (77.06%)	2,078 (53.75%)	1,188 (30.73%)	789 (20.41%)	
Weight, kg	49.37 ± 5.84	56.47 ± 6.59	63.33 ± 7.07	73.39 ± 9.91	<0.001
Height, m	1.62 ± 0.08	1.64 ± 0.09	1.67 ± 0.08	1.68 ± 0.08	<0.001
BMI, kg/m^2^	18.78 ± 1.35	20.92 ± 1.17	22.77 ± 1.27	26.00 ± 2.51	<0.001
WC, cm	67.25 ± 4.99	73.15 ± 5.25	78.79 ± 5.17	86.69 ± 6.82	<0.001
ALT, IU/L	13.00 (11.00–17.00)	15.00 (12.00–19.00)	18.00 (14.00–23.00)	24.00 (18.00–34.00)	<0.001
AST, IU/L	16.00 (13.00–19.00)	16.00 (13.00–20.00)	17.00 (14.00–21.00)	20.00 (16.00–24.00)	<0.001
GGT, IU/L	12.00 (10.00–15.00)	13.00 (10.00–18.00)	16.00 (13.00–24.00)	23.00 (16.00–34.00)	<0.001
HDL-C, mg/dl	65.00 (55.90–75.90)	59.00 (50.30–69.00)	52.00 (44.00–61.00)	44.20 (38.00–51.60)	<0.001
TC, mg/dl	184.33 ± 30.62	195.27 ± 31.73	201.65 ± 31.99	211.56 ± 33.26	<0.001
TG, mg/dl	38.00 (29.00–50.00)	56.00 (43.00–72.00)	77.00 (58.00–100.00)	121.00 (87.00–166.00)	<0.001
HbA1c, %	5.12 ± 0.30	5.14 ± 0.31	5.17 ± 0.32	5.25 ± 0.34	<0.001
FPG, mg/dl	88.50 ± 6.80	91.76 ± 6.88	94.42 ± 6.61	97.19 ± 6.52	<0.001
TyG index	7.41 ± 0.45	7.85 ± 0.40	8.20 ± 0.42	8.68 ± 0.50	<0.001
SBP, mmHg	105.65 ± 12.18	111.49 ± 13.25	116.94 ± 13.37	123.91 ± 14.57	<0.001
DBP, mmHg	65.43 ± 8.47	69.35 ± 9.41	73.31 ± 9.52	78.23 ± 10.02	<0.001
Fatty liver	22 (0.57%)	140 (3.62%)	592 (15.31%)	1,987 (51.40%)	<0.001
Habit of exercise	621 (16.06%)	755 (19.53%)	726 (18.78%)	607 (15.70%)	<0.001
Drinking status					<0.001
None	3,356 (86.81%)	3,002 (77.65%)	2,754 (71.24%)	2,693 (69.66%)	
Light	288 (7.45%)	449 (11.61%)	521 (13.48%)	500 (12.93%)	
Moderate	179 (4.63%)	314 (8.12%)	419 (10.84%)	448 (11.59%)	
Heavy	43 (1.11%)	101 (2.61%)	172 (4.45%)	225 (5.82%)	
Smoking status					<0.001
Non	2,987 (77.26%)	2,461 (63.66%)	1,924 (49.77%)	1,659 (42.91%)	
Past	391 (10.11%)	651 (16.84%)	923 (23.87%)	987 (25.53%)	
Current	488 (12.62%)	754 (19.50%)	1,019 (26.36%)	1,220 (31.56%)	
Normal-high BP values	474 (12.26%)	849 (21.96%)	1,288 (33.32%)	1,805 (46.69%)	
Hypertension	41 (1.06%)	127 (3.29%)	247 (6.39%)	549 (14.2%)	

Data were mean ± SD or median (interquartile range) for skewed variables or numbers (proportions) for categorical variables. BMI, body mass index; WC, waist circumference; ALT, alanine aminotransferase; AST, aspartate aminotransferase; GGT, gamma-glutamyl transferase; HDL-C, high-density lipoprotein; TC, total cholesterol; TG, triglyceride; HbA1c, hemoglobin A1c; FPG, fasting plasma glucose; TyG index, triglyceride-glucose index; SBP, systolic blood pressure; DBP, diastolic blood pressure; TyG-BMI, triglyceride glucose-body mass index; Q1, Q2, Q3, and Q4 are quartiles of the TyG-BMI.

### Multivariate analysis between TyG-BMI and normal-high BP values or HTN

Four multivariate logistic regression models were developed to assess the independent effects of TyG-BMI on normal-high BP values and HTN (Table 2). As can be seen, the degree of associations between TyG-BMI and normal-high BP values and HTN changed slightly in each of the four stepwise adjusted models, while the direction of the relevant associations remained consistent throughout; and in the fully adjusted model (Model 4), when TyG-BMI was used as a continuous variable, the OR value corresponding to the association between TyG-BMI and normal-high BP values/HTN was 2.35; when TyG-BMI was used as a categorical variable, compared with the lowest quartile (Q1) of TyG-BMI the regression coefficient for the association of the highest quartile (Q4) of TyG-BMI with normal-high BP values increased by 426%, while the regression coefficient for the association with HTN increased by 527%; the linear trend test (*P*-trend <0.0001) further suggested that there may be a linear relationship between TyG-BMI and normal-high BP values and HTN. In addition, we further compared the strength of the correlation between BMI, TyG index, TyG-BMI and normal-high BP values and HTN (Supplementary Table S3). After standardized OR values (unified *Z* transformation and then incorporated into the multivariate regression model), the results showed that TyG-BMI had the highest standardized OR value in evaluating HTN and normal-high BP values, followed by BMI, and finally TyG index [model 4: HTN (OR): TyG-BMI 2.35 > BMI 1.96 > TyG index 1.51; normal-high BP(OR): TyG-BMI 2.35 > BMI 1.94 > TyG index 1.31].

**Table 2:** Multivariable-adjust ORs and 95% CI of the TyG-BMI index quartiles associated with normal-high BP values and hypertension.

Odds ratios (95% confidence interval)					
	Crude model	Model 1	Model 2	Model 3	Model 4
Hypertension					
TyG-BMI (Per SD increase)	2.35 (2.21, 2.50)	2.32 (2.17, 2.48)	2.21 (2.04, 2.39)	2.37 (2.15, 2.61)	2.35 (2.13, 2.60)
TyG-BMI (Quartile)					
Q 1	Ref	Ref	Ref	Ref	Ref
Q 2	3.17 (2.22, 4.52)	2.44 (1.71, 3.50)	2.38 (1.66, 3.40)	2.24 (1.56, 3.22)	2.24 (1.56, 3.23)
Q 3	6.37 (4.56, 8.89)	4.22 (2.99, 5.94)	3.81 (2.70, 5.38)	3.52 (2.46, 5.03)	3.49 (2.44, 4.98)
Q 4	15.44 (11.20, 21.28)	10.15 (7.28, 14.16)	7.52 (5.33, 10.63)	6.45 (4.42, 9.39)	6.27 (4.30, 9.15)
*P*-trend	<0.001	<0.001	<0.001	<0.001	<0.001
Normal-high BP values					
TyG-BMI (Per SD increase)	2.38 (2.28, 2.48)	2.11 (2.02, 2.21)	2.10 (2.00, 2.21)	2.33 (2.18, 2.49)	2.35 (2.19, 2.51)
TyG-BMI (Quartile)					
Q 1	Ref	Ref	Ref	Ref	Ref
Q 2	2.08 (1.84, 2.35)	1.71 (1.51, 1.94)	1.70 (1.50, 1.93)	1.66 (1.46, 1.89)	1.68 (1.48, 1.92)
Q 3	3.91 (3.47, 4.39)	2.79 (2.46, 3.16)	2.71 (2.39, 3.07)	2.66 (2.32, 3.05)	2.70 (2.36, 3.10)
Q 4	8.44 (7.50, 9.50)	5.85 (5.16, 6.64)	5.26 (4.59, 6.03)	5.23 (4.44, 6.15)	5.26 (4.46, 6.20)
*P*-trend	<0.001	<0.001	<0.001	<0.001	<0.001

BMI, body mass index; TyG index, triglyceride and glucose index; TyG-BMI, triglyceride glucose-body mass index; CI, confidence interval; OR, odds ratios; Ref, reference. Model 1 adjusted for sex, age and height. Model 2 adjusted for Model 1 + fatty liver, habit of exercise, drinking status and smoking status. Model 3 adjusted for Model 2 + HDL-C, TC, TG, HbA1c and FPG. Model 4 adjusted for Model 3 + ALT, AST and GGT.

### Dose-response relationship between TyG-BMI and SBP, DBP

**Figure 2:**
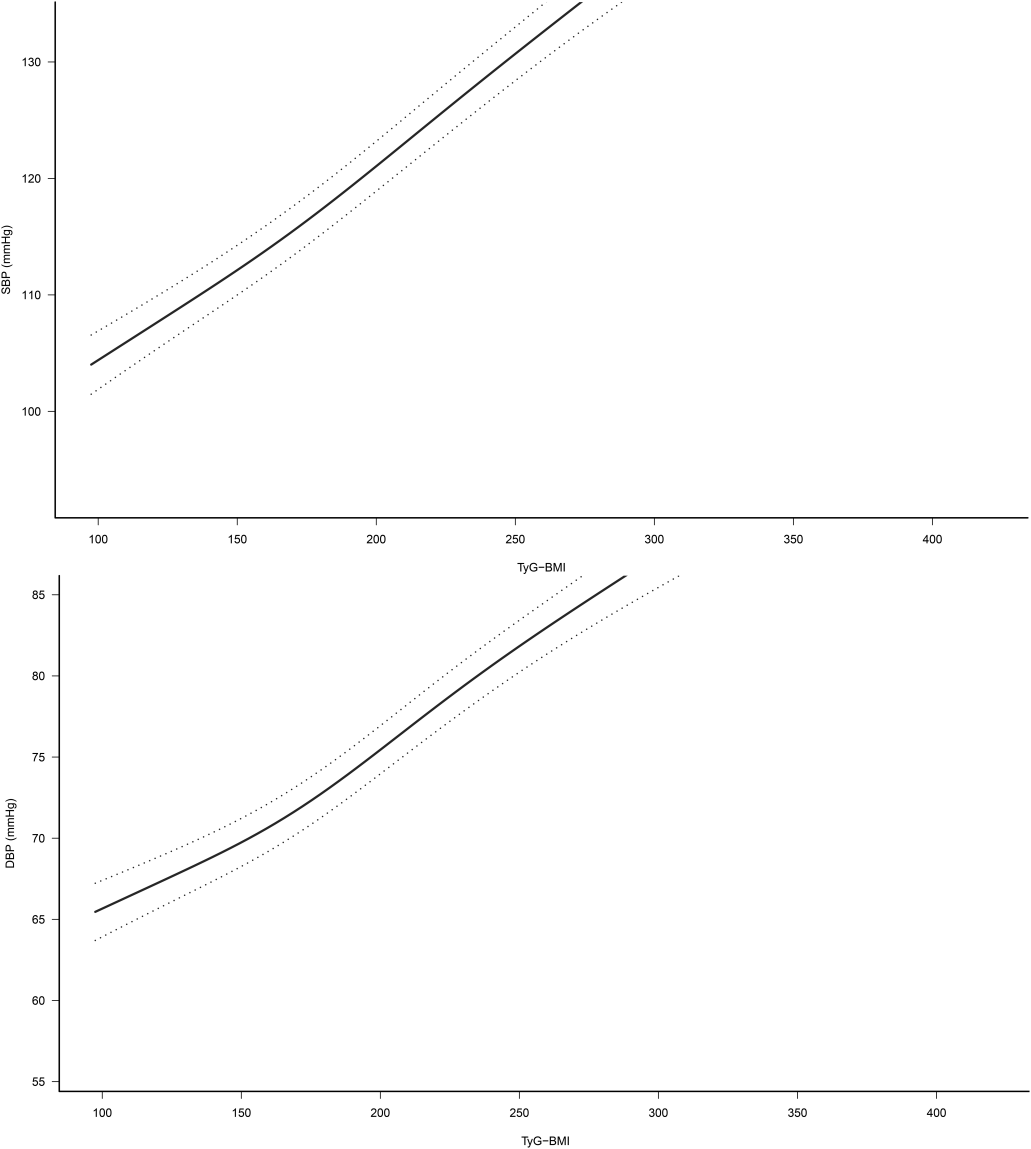
Dose-response relationship between TyG-BMI and SBP/DBP (95% confidence interval was shown by dashed line). Adjusted for sex, age, height, fatty liver, habit of exercise, drinking status, smoking status, HDL-C, TC, TG, HbA1c, FPG, ALT, AST and GGT. TyG-BMI, triglyceride glucose-body mass index.

Based on model 4, we further fitted the dose-response relationship between TyG-BMI and SBP, DBP by natural smoothing splines. The results showed that after fully adjusting the confounding factors, TyG-BMI was linearly positively correlated with SBP and DBP (Figure 2). This result further supported the linear trend of TyG-BMI and HTN/normal-high BP values.

### Accuracy of TyG-BMI, BMI, and TyG index for identifying normal-high BP values or HTN

**Figure 3:**
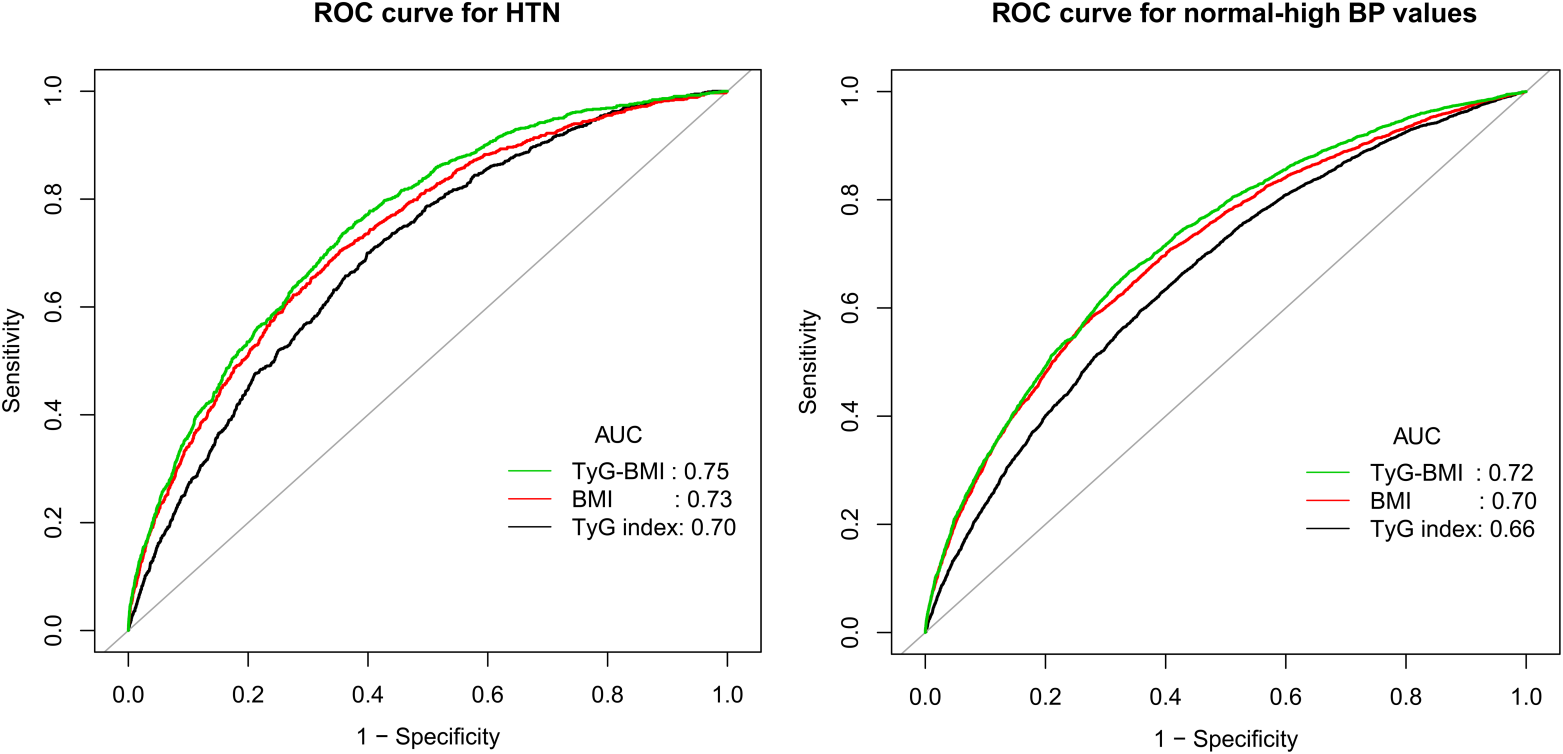
Receiver operating characteristic curve analyzes the value of BMI, TyG index and TyG-BMI in identifying HTN/normal-high BP values. BMI, body mass index; TyG index, triglyceride-glucose index; TyG-BMI, triglyceride glucose-body mass index; AUC, the area under the curve; HTN, hypertension.

To compare the ability of TyG-BMI, BMI, and TyG index for identifying normal-high BP values and HTN, we plotted the ROC curves of the three parameters (Figure 3) and calculated the corresponding AUC values, optimal cut-off values, specificity, and sensitivity (Table 3). The results showed that the AUCs of the three parameters were all greater than 0.6, among them, TyG-BMI (AUC: 0.72 and 0.75) had the highest ability to recognize normal-high BP values/HTN, followed by BMI (AUC: 0.70 and 0.73), and finally TyG index (AUC: 0.66 and 0.70) (All Delong *P *< 0.001). In addition, we calculated the optimal cut-off values of TyG-BMI for identifying normal-high BP values and HTN as 177.46 and 185.19, respectively.

**Table 3:** Areas under the receiver operating characteristic curves for each evaluated parameters in identifying normal-high BP values and hypertension.

	AUC	95% CI low	95% CI upp	Best threshold	Specificity	Sensitivity
Hypertension						
TyG index*,**	0.70	0.68	0.72	8.14	0.60	0.70
BMI*	0.73	0.72	0.75	22.79	0.65	0.70
TyG-BMI	0.75	0.74	0.77	185.19	0.64	0.74
Normal-high BP values						
TyG index*,**	0.66	0.65	0.67	8.10	0.64	0.59
BMI*	0.70	0.69	0.71	22.65	0.72	0.58
TyG-BMI	0.72	0.71	0.73	177.46	0.66	0.67

BMI, body mass index; TyG index, triglyceride and glucose index; TyG-BMI, triglyceride glucose-body mass index; AUC, the area under the curve.

* *P *< 0.001, DeLong test was used to compare the AUC of TyG-BMI and TyG index/BMI.

** *P *< 0.001, DeLong test was used to compare the AUC of BMI and TyG index.

### Subgroup analysis by adjusted potential effect confounders

To explore whether there were differences in the association between TyG-BMI and normal-high BP values/HTN in different populations, we also performed subgroup analyses. As shown in Table 4, we found an interaction effect only in the age subgroup; it is worth mentioning that the correlation between TyG-BMI and normal-high BP values/HTN gradually decreased with aging. In contrast, we did not find significant interactions in the subgroups of sex, BMI, drinking status, and smoking status (*P-*interaction >0.05).

**Table 4:** Stratified association between TyG-BMI and HTN/normal-high BP values by age, sex, BMI, smoking status, and drinking status.

	Hypertension		Normal-high BP values	
	Adjusted OR (95% CI)	*P-interaction*	Adjusted OR (95% CI)	*P-interaction*
Age (years)		0.001		<0.001
<30	3.75 (1.85, 7.61)		3.35 (2.39, 4.70)	
30–44	2.57 (2.28, 2.91)		2.60 (2.40, 2.82)	
45–59	2.11 (1.87, 2.39)		2.03 (1.86, 2.22)	
≥60	1.59 (1.19, 2.14)		1.59 (1.28, 1.98)	
Sex		0.735		0.157
Women	2.32 (2.02, 2.65)		2.44 (2.24, 2.65)	
Men	2.38 (2.12, 2.67)		2.27 (2.09, 2.46)	
BMI, kg/m^2^		0.796		0.581
<25	2.40 (2.01, 2.86)		2.24 (2.05, 2.46)	
≥25	2.46 (2.08, 2.91)		2.14 (1.82, 2.52)	
Drinking status		0.086		0.456
None	2.33 (2.10, 2.59)		2.36 (2.19, 2.53)	
Light	2.80 (2.28, 3.45)		2.40 (2.07, 2.77)	
Moderate	2.30 (1.86, 2.85)		2.12 (1.81, 2.48)	
Heavy	1.86 (1.39, 2.48)		2.61 (2.02, 3.39)	
Smoking status		0.627		0.178
Non	2.37 (2.11, 2.66)		2.43 (2.25, 2.62)	
Past	2.20 (1.85, 2.61)		2.27 (2.02, 2.55)	
Current	2.41 (2.07, 2.81)		2.20 (1.99, 2.44)	

BMI, body mass index; TyG-BMI, triglyceride glucose-body mass index; CI, confidence interval, OR, odds ratios. Adjusted for sex, age and height, fatty liver, habit of exercise, drinking status, smoking status, HDL-C, TC, TG, HbA1c, FPG, ALT, AST and GGT. In each case, the model is not adjusted for the stratification variable.

## Discussion

In the current epidemiological survey based on the non-diabetic population, we found that (1) BMI, TyG index, and TyG-BMI were all significantly and independently positively associated with normal-high BP values and HTN in the non-diabetic population; compared with BMI and TyG index alone, TyG-BMI had higher recognition ability for normal-high BP values and HTN. (2) TyG-BMI was more strongly associated with normal-high BP values or HTN in the youth population.

In the past two decades, slightly elevated BP within the normal range has attracted much attention due to its adverse clinical effects (8, 9). Normal-high BP values have been reported to affect approximately 25%–50% of adults worldwide and significantly increase the risk of developing HTN (10). In addition, the risk of cardiovascular disease increases further with increasing BP, and previous studies have shown that the risk of cardiovascular disease was significantly higher in people with BP in the 120–129/80–84 mmHg range and 130–139/85–89 mmHg range than those with the BP in the 120/80 mmHg range (38–40). Therefore, early detection and intervention of normal-high BP values are important to prevent HTN.

The pathogenesis of HTN is complex, and normal-high BP values and HTN individuals are often accompanied by abnormal glucose and lipid homeostasis; it has been reported that about 40% of HTN patients have dyslipidemia and impaired fasting glucose (41–43), and these abnormal glucose and lipid metabolisms will further increase the risk of cardiovascular disease. IR is a key mechanism of glycolipid metabolism (16) and an important precursor to the early stages of the disease. Generally speaking, the physiological function of insulin to lower blood glucose is somewhat limited when IR occurs, and the body then secretes as much insulin as possible. In addition, excessive secretion of insulin tends to produce hyperinsulinemia, which becomes a potential disease hazard (44, 45); hyperinsulinemia can contribute to increased renal tubular sodium reabsorption, sympathetic excitation, accelerated heart rate, increased vascular resistance, dyslipidemia, and narrowing of atherosclerosis, which in turn can increase intracellular calcium ion concentration and sensitivity to elevating substances, leading to the development of HTN (16, 46).

TyG index, a combined marker containing TG and FPG, has previously been extensively studied and is considered an alternative marker for IR (31, 47, 48). In a recent study, Professor Zeng and his team found that the TyG index can also be used to identify individuals at high risk for normal-high BP values (49), and their findings further suggested that IR was strongly associated with normal-high BP values. BMI is the most important anthropometric measure commonly used in clinical practice to evaluate obesity and is a recognized predictor of HTN (50). Obesity is often accompanied by an elevated prevalence of HTN; conversely, weight control is effective in reducing BP (50, 51). TyG-BMI is a newly developed IR surrogate in recent years, which is the product of BMI and TyG index. In one of the earliest TyG-BMI-related studies, Er et al. found that TyG-BMI could better reflect IR compared with lipid parameters, lipid ratios, glucose parameters, TyG index, and obesity-related parameters (22). Several subsequent studies further found that TyG-BMI was also strongly associated with a variety of metabolic diseases (23–27). In March 2021, Raimi et al. analyzed the relationship between TyG-BMI and MS, and they pointed out that the product combination of BMI and TyG index improved the ability to identify MS (23); in May of the same year, in a cross-sectional study by Li et al., they found that TyG-BMI had moderate discriminatory power in distinguishing HTN from hyperuricemia (26). In addition, some observational studies have further found that TyG-BMI also has the potential to predict the risk of NAFLD and diabetes, especially in young and middle-aged and non-obese people (24, 27). Similarly, in a longitudinal cohort study with a median follow-up time of 3.1 years, Jiang et al. also found similar results, in which higher TyG-BMI levels significantly increased the risk of prediabetes (26). These findings all suggested that TyG-BMI has a certain application value in metabolic diseases. However, there are relatively few studies on the associations between TyG-BMI and normal-high BP values and HTN, and in a recent cross-sectional study of Chinese lean-weight adults by Zeng et al., they found that TyG-BMI showed a strong positive correlation with normal-high BP values (49), which was similar to the results of our subgroup analysis, but in further analysis, we found that the associations between TyG-BMI and normal-high BP values/HTN were not significantly different between obese and non-obese groups, further research is needed. Compared with the research of Zeng et al., we further calculated the OR value and 95% CI of the corresponding normal-high BP values and HTN after Z-conversion of BMI, TyG index and TyG-BMI. Before and after the adjustment of the model, among the three parameters, the TyG-BMI index had the strongest correlation with normal-high BP values and HTN, followed by BMI, and finally TyG index. In addition, it is worth mentioning that the current study also found a similar trend in ROC analysis: among the three parameters, TyG-BMI has the highest accuracy in identifying normal-high BP values and HTN, followed by BMI, and finally TyG index. These results together suggested that, after a further combination of TyG index and BMI, BMI-related components may greatly improve the assessment/recognition ability for HTN and normal-high BP values. Based on the background of the current research (15–17), the reason for this result may be closely related to IR, and the combination of the TyG index and obesity index BMI improved the ability to identify IR (22), thereby enhancing the ability to identify HTN/normal-high BP values. In addition, we also constructed natural smoothing splines and found that TyG-BMI was linearly positively correlated with SBP and DBP. This further supported the linear trend of TyG-BMI with HTN/normal-high BP values. To my knowledge, the current study revealed for the first time a linear association between TyG-BMI and SBP/DBP. These new findings may be able to provide new strategies and approaches for the prevention of normal-high BP values and HTN in different populations.

We also found some interesting results in our subgroup analysis: compared with other age groups, the correlation between TyG-BMI and normal-high BP values and HTN was relatively higher in young people. We speculated that the reason for this result may be related to the increasing poor dietary habits as well as lifestyle habits of young people. It has been shown that obesity during childhood and adolescence greatly increases the risk of developing HTN in adulthood (52). Not only that, some poor lifestyles such as a high-salt diet, sedentary lifestyle, excessive alcohol consumption, lack of exercise, chronic mental stress, and sleep deprivation are problems faced by contemporary young people, which greatly contribute to the prevalence of HTN (53). In addition, longitudinal studies have reported that high BP levels measured in childhood and adolescence often transform into stable hypertension in adulthood, with significant and substantial epidemiological implications and clinical relevance, a phenomenon known as “BP tracking” (54–56).

### Strengths and limitations

A significant advantage of this work is that the association between TyG-BMI and normal-high BP values or HTN in the general population was revealed for the first time through a rigorous statistical analysis strategy supported by large sample data.

This study also has some limitations that have to be mentioned: (1) The relevant parameters for evaluating insulin were not included in the current dataset, so HOMA-IR was not further evaluated for result verification. (2) The study population was Japanese, so the external applicability of the findings needs to be confirmed by further studies. (3) The current study was a secondary analysis of the previous research dataset, and all the identifiable information in the analyzed data set has been deleted (including name and registration time), so we can't further evaluate the association between TyG-BMI and HTN/normal-high BP values in different years. Additionally, the covariates contained in the dataset were limited, so it was inevitably a certain residual confounding in the current study (57). (4) The cross-sectional study design limited the ability to determine causality, so we also could not determine the predictive value of TyG-BMI for normal-high BP values or HTN. (5) Because the NAGALA study set more exclusion conditions in the enrollment of subjects, this may result in more patients diagnosed with HTN not being included in the current study, which further leads to a lower prevalence of HTN in the current study. (6) Serum uric acid (UA) is an important metabolic factor that is easily overlooked; in fact, UA is closely related to the composition of TyG-BMI and HTN (58, 59), which may be an important mediating factor between TyG-BMI and normal-high BP values/HTN. However, since the parameter UA is not included in the current research data set, this potential mechanism needs to be evaluated in further research.

## Conclusion

In the non-diabetic population, TyG-BMI was significantly and positively associated with both normal-high BP values and HTN; TyG-BMI has higher values for both normal-high BP values and HTN identification compared to BMI and TyG index alone. TyG-BMI has the potential to be a cost-effective monitoring indicator as well as a non-invasive assessment method in the individual stratified management of normal-high BP value and may serve as an accessible complementary monitoring method in the future risk stratification management of HTN.

## Data Availability

The original contributions presented in the study are included in the article/**Supplementary Material**, further inquiries can be directed to the corresponding author.
