# Supplementary material for: Associations between TyG-BMI and normal-high blood pressure values and hypertension: cross-sectional evidence from a non-diabetic population
Source: Front Cardiovasc Med. 2023 Apr 24;10:1129112. doi: 10.3389/fcvm.2023.1129112 (PMC10164981; doi:10.3389/fcvm.2023.1129112)
Supplement: Supplementary file 1 [file Table1.docx]

Supplementary Table 1: Screening for collinearity of TyG-BMI with other covariates with normal-high blood pressure values as dependent variable.

|  | VIF | | | | |
| --- | --- | --- | --- | --- | --- |
|  | Step 1 | Step 2 | Step 3 | Step 4 | Step 5 |
| TyG-BMI | 422.1 | 151.1 | 8.9 | 7.4 | 2.8 |
| Sex | 3.2 | 3.2 | 3.2 | 3.2 | 3.2 |
| Age, years | 1.4 | 1.4 | 1.4 | 1.3 | 1.3 |
| Weight, kg | 177.4 | 147.8 | NA | NA | NA |
| Height, cm | 54.6 | 45.3 | 2.8 | 2.8 | 2.4 |
| BMI | 269.5 | NA | NA | NA | NA |
| WC | 6 | 5.9 | 5.9 | 5.3 | NA |
| Fatty liver | 1.6 | 1.6 | 1.6 | 1.6 | 1.6 |
| Habit of exercise | 1 | 1 | 1 | 1 | 1 |
| ALT, IU/L | 4.2 | 4.1 | 4.1 | 4.1 | 4.1 |
| AST, IU/L | 3.3 | 3.3 | 3.3 | 3.3 | 3.3 |
| GGT, IU/L | 1.5 | 1.5 | 1.5 | 1.5 | 1.5 |
| HDL-C, mg/dL | 1.9 | 1.9 | 1.9 | 1.8 | 1.8 |
| TC, mg/dL | 1.5 | 1.5 | 1.5 | 1.5 | 1.5 |
| TG, mg/dL | 6.4 | 5.6 | 5.3 | 2.5 | 2.1 |
| HbA1c, % | 1.3 | 1.3 | 1.3 | 1.3 | 1.2 |
| FPG, mg/dL | 1.6 | 1.6 | 1.6 | 1.5 | 1.5 |
| TyG index | 63.4 | 27.9 | 8.4 | NA | NA |
| Drinking status | 1.3 | 1.3 | 1.3 | 1.3 | 1.3 |
| Smoking status | 1.4 | 1.4 | 1.4 | 1.4 | 1.4 |

Abbreviations: VIF: Variance inflation factor; Other abbreviations as in Table ​1.

Note: VIF = 1/(1-R^2^).

Supplementary Table 2: Screening for collinearity of TyG-BMI with other covariates with hypertension as dependent variable.

|  | VIF | | | | |
| --- | --- | --- | --- | --- | --- |
|  | Step 1 | Step 2 | Step 3 | Step 4 | Step 5 |
| TyG-BMI | 422.1 | 151.1 | 8.9 | 7.4 | 2.8 |
| Sex | 3.2 | 3.2 | 3.2 | 3.2 | 3.2 |
| Age, years | 1.4 | 1.4 | 1.4 | 1.3 | 1.3 |
| Weight, kg | 177.4 | 147.8 | NA | NA | NA |
| Height, cm | 54.6 | 45.3 | 2.8 | 2.8 | 2.4 |
| BMI | 269.5 | NA | NA | NA | NA |
| WC | 6 | 5.9 | 5.9 | 5.3 | NA |
| Fatty liver | 1.6 | 1.6 | 1.6 | 1.6 | 1.6 |
| Habit of exercise | 1 | 1 | 1 | 1 | 1 |
| ALT, IU/L | 4.2 | 4.1 | 4.1 | 4.1 | 4.1 |
| AST, IU/L | 3.3 | 3.3 | 3.3 | 3.3 | 3.3 |
| GGT, IU/L | 1.5 | 1.5 | 1.5 | 1.5 | 1.5 |
| HDL-C, mg/dL | 1.9 | 1.9 | 1.9 | 1.8 | 1.8 |
| TC, mg/dL | 1.5 | 1.5 | 1.5 | 1.5 | 1.5 |
| TG, mg/dL | 6.4 | 5.6 | 5.3 | 2.5 | 2.1 |
| HbA1c, % | 1.3 | 1.3 | 1.3 | 1.3 | 1.2 |
| FPG, mg/dL | 1.6 | 1.6 | 1.6 | 1.5 | 1.5 |
| TyG index | 63.4 | 27.9 | 8.4 | NA | NA |
| Drinking status | 1.3 | 1.3 | 1.3 | 1.3 | 1.3 |
| Smoking status | 1.4 | 1.4 | 1.4 | 1.4 | 1.4 |

Abbreviations: VIF: Variance inflation factor; Other abbreviations as in Table ​1.

Note: VIF = 1/(1-R^2^).

Supplementary Table 3: Multivariable-adjust ORs and 95%CI of the BMI, TyG index, and TyG-BMI associated with normal-high blood pressure values and hypertension.

| Odds ratios (95% confidence interval) | | | | | |
| --- | --- | --- | --- | --- | --- |
|  | Crude model | Model 1 | Model 2 | Model 3 | Model 4 |
| Hypertension |  |  |  |  |  |
| BMI (Per SD increase) | 2.16 (2.04,2.29) | 2.17 (2.04,2.32) | 2.02 (1.88,2.18) | 1.97 (1.83,2.13) | 1.96 (1.81,2.12) |
| TyG index (Per SD increase) | 2.02 (1.89,2.16) | 1.75 (1.62,1.88) | 1.50 (1.39,1.63) | 1.53 (1.25,1.88) | 1.51 (1.23,1.86) |
| TyG-BMI (Per SD increase) | 2.35 (2.21, 2.50) | 2.32 (2.17, 2.48) | 2.21 (2.04, 2.39) | 2.37 (2.15,2.61) | 2.35 (2.13,2.60) |
| Normal-high blood pressure values | | | | | |
| BMI (Per SD increase) | 2.27 (2.17,2.37) | 2.05 (1.96,2.14) | 1.99 (1.90,2.09) | 1.93(1.84,2.04) | 1.94(1.84,2.05) |
| TyG index (Per SD increase) | 1.82 (1.75,1.89) | 1.52 (1.45,1.59) | 1.40 (1.34,1.47) | 1.29(1.16,1.40) | 1.31(1.17,1.46) |
| TyG-BMI (Per SD increase) | 2.38 (2.28, 2.48) | 2.11 (2.02, 2.21) | 2.10 (2.00, 2.21) | 2.33 (2.18,2.49) | 2.35 (2.19,2.51) |

Abbreviations: BMI: body mass index; TyG index: triglyceride and glucose index; TyG-BMI: triglyceride glucose-body mass index; CI: confidence interval; OR: odds ratios; Ref, reference.

Model 1 adjusted for sex, age and height;

Model 2 adjusted for Model 1 + fatty liver, habit of exercise, drinking status and smoking status;

Model 3 adjusted for Model 2 + HDL, TC, TG, HbA1c and FPG;

Model 4 adjusted for Model 3 + ALT, AST and GGT.
